# Atherogenic index of plasma is associated with the severity of Hidradenitis Suppurativa: a case-control study

**DOI:** 10.1186/s12944-020-01377-6

**Published:** 2020-08-29

**Authors:** José L. Hernández, Cristina Baldeón, Ana E. López-Sundh, J. Gonzalo Ocejo-Vinyals, Ricardo Blanco, Marcos A. González-López

**Affiliations:** 1 Division of Internal Medicine, Hospital Universitario Marqués de Valdecilla, University of Cantabria, IDIVAL, Santander, Spain; 2 Division of Internal Medicine, Hospital Universitario Marqués de Valdecilla, University of Cantabria, Santander, Spain; 3 Division of Dermatology, Hospital Universitario Marqués de Valdecilla, University of Cantabria, Santander, Spain; 4 Division of Immunology, Hospital Universitario Marqués de Valdecilla, University of Cantabria, IDIVAL, Santander, Spain; 5 Division of Rheumatology, Hospital Universitario Marqués de Valdecilla, University of Cantabria, IDIVAL, Santander, Spain; 6 Division of Dermatology, Hospital Universitario Marqués de Valdecilla, IDIVAL, University of Cantabria, 39008 Santander, Spain

**Keywords:** Hidradenitis suppurativa, Atherogenic indexes, Atherogenic index of plasma, Lipids

## Abstract

**Background:**

Hidradenitis suppurativa (HS) is a chronic inflammatory disease associated with several comorbidities and vascular risk factors, such as dyslipidemia. The present study aimed to assess the possible associations between the lipid profile and atherogenic indexes and the severity of HS.

**Methods:**

This case-control study enrolled 78 HS patients and 62 healthy controls. Classic lipid profile and lipoprotein ratios, including the atherogenic index of plasma (AIP), were evaluated. The severity of HS was measured by the HS Physician Global Assessment (PGA).

**Results:**

HS-patients had lower serum total cholesterol and HDL-C levels and higher AIP than the control group. AIP was positively correlated to BMI, waist circumference, systolic and diastolic blood pressure, LDL-C, triglycerides, non-HDL-C, ApoB, HOMA, and hs-CRP and negatively to HDL-C and ApoA1. For the overall lipid profile, only AIP was related to a more severe HS (PGA ≥ 3) after controlling for age, sex, BMI, insulin resistance (IR), active smoking, and statin use (*r =* 0.268; *p* = 0.023). Multiple logistic regression adjusted for age, sex, BMI, IR, smoking status and statin use, showed that AIP ≥ 0.11 was significantly associated with the severity of HS (OR, 4.38; CI 95%, 1.09–17.50; *p* = 0.037).

**Conclusions:**

In conclusion, these results showed that AIP is significantly and independently associated with HS severity.

## Background

Hidradenitis suppurativa (HS) is a recurrent chronic inflammatory disease presenting with painful, suppurating lesions affecting the apocrine gland-rich regions of the skin. Prevalence is estimated from 0.05 to 4% across series, and this condition is associated with severe impairment of the quality of life [1]. HS has been related to certain comorbidities and metabolic disorders such as type 2 diabetes mellitus (T2DM) and metabolic syndrome, both characterized by the common link of insulin resistance (IR) [2–4]. Furthermore, an increased prevalence of vascular risk factors, subclinical atherosclerosis, and a significant risk for major adverse cardiovascular events have also been reported in HS-patients [5–7].

The precise inflammatory mechanism underlying HS is still not fully elucidated, although genetic susceptibility, mechanical stress, obesity, microbiome, and environmental and hormonal factors have been involved [8, 9]. Moreover, in HS-patients, the presence in the affected skin areas and/or in the serum of increased expression of several cytokines, such as interleukin (IL)-1β, IL-6, IL-17, interferon-gamma, and tumor necrosis factor-alpha (TNF-α) suggest a role for the autoinflammation in HS pathogenesis [9].

Atherogenic dyslipidemia, i.e. the combined occurrence of high fasting blood concentrations of triglycerides (TG) and low levels of high-density lipoprotein cholesterol (HDL-C), is frequent in patients with HS [10]. Nevertheless, data on the relationship between the values of nontraditional lipid profiles (lipoprotein ratios), including non-HDL-C, total cholesterol (TC)/HDL-C, LDL-C/HDL-C, non-HDL-C/HDL-C (atherogenic index, AI), atherogenic index of plasma (AIP) and Apolipoprotein B/Apolipoprotein A1 (ApoB/ApoA1) ratio, and the severity of HS are lacking. Compared with single lipid parameters, these comprehensive lipid ratios are considered to be better predictors for coronary artery disease [11, 12], mainly the AIP [13–15]. This index has shown a good correlation with smaller LDL-C particles and also with increased fractional esterification rate for cholesterol in plasma, and it is a strong and independent predictor factor for coronary disease [16–18]. Noteworthy, AIP has also been associated with raised serum C-reactive protein (CRP) levels, suggesting a lipid-driven immune-inflammatory link [19].

Taking into account the above considerations, the present study aimed to assess the possible associations between the lipid profile and atherogenic indexes and the severity of HS.

## Methods

### Study population

**Fig. 1:**
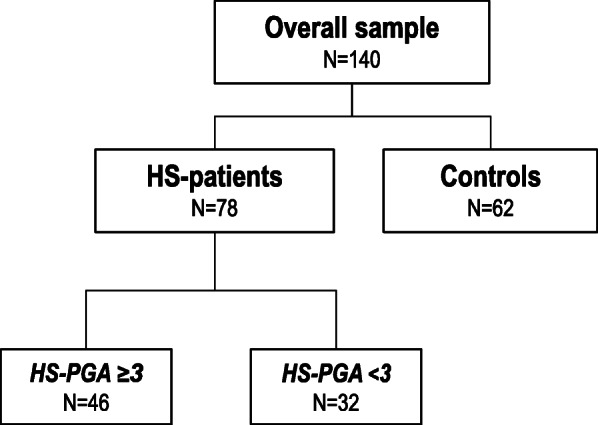
Flow chart of the study subjects. Footnote: *HS-PGA: Hidradenitis Suppurativa Physician Global Assessment*

In this cross-sectional case-control study, 78 patients with HS and 62 age- and gender-similar controls were included. HS-patients were recruited from the Dermatology outpatient clinic of a teaching tertiary-care hospital in Northern Spain. The control group was set up with hospital medical staff and subjects who attended the Dermatology clinic due to skin disorders other than HS, such as epithelioma, warts, or melanocytic nevus. The flow-chart of the study subjects is shown in Fig. 1. The research protocol was approved by the local Ethics Committee (internal code: 2013.267) and all study procedures were done under the ethical principles of the Declaration of Helsinki. All the participants provided written informed consent.

For the present study, exclusion criteria were as follows: age < 18 years, documented history of major adverse cardiovascular events, chronic kidney or liver diseases, and the presence of concomitant inflammatory cutaneous or systemic disorders.

### Data collection and working definitions

The following variables were collected in all the participants: demographic features, past medical history, current and prior systemic therapy, traditional cardiovascular risk factors (defined as previously reported) [2], weight, height, body mass index (BMI), waist circumference (WC), systolic blood pressure (BP) and diastolic BP. Information about HS duration was collected in all the patients.

The HS Physician Global Assessment (HS-PGA) and the Hurley scale were used to assessing the severity of HS. Thus, HS was classified as moderate-severe-very severe (PGA ≥ 3) and as minimal-mild HS (PGA < 3) according to HS-PGA [2].

Metabolic syndrome was diagnosed according to the criteria proposed by the National Cholesterol Education Program’s Adult Treatment Panel III (ATP III) [20]. AIP is defined as the base 10 logarithm of the ratio of the concentration of TG to HDL-C with TG and HDL-C expressed in mmol/L [14].

### Laboratory assessments

Blood samples were drawn after an overnight fast, and serum TC, LDL-C, HDL-C, TG, ApoB, ApoA1 and lipoprotein (a) (Lpa) were analyzed, as well as the following atherogenic indexes: TC/HDL-C; LDL-C/HDL-C; non-HDL-C/HDL-C; ApoB/ApoA1, and AIP. Moreover, the homeostatic model assessment for IR (HOMA-IR) was calculated as fasting insulin level (μIU/ml) x fasting glucose level (mg/dL)/405. A value greater than 2.5 is consistent with IR [2].

### Statistical analysis

Results were expressed as numbers (percentage), mean ± standard deviation (SD) or median and interquartile range (IQR), as appropriate. Student’s T-test or Mann-Whitney U-test was used to compare quantitative variables and Chi-squared or Fisher test, to compare qualitative data, as appropriate. The Spearman correlations coefficients were calculated to assess the relationship between AIP and demographic and laboratory parameters in cases and controls. The patients were divided into two groups according to disease severity based on the PGA score (< 3 and ≥ 3). A cut-off point for AIP ≥0.11 for both sexes, based on previously published data, has been considered for the analysis [21].To assess the potential association between AIP and HS severity, forward stepwise multiple logistic regression models adjusted for confounder variables were built. The strength of the association between the study parameters and HS severity was evaluated via the odds ratio (OR) and 95% confidence interval (CI). Finally, ROC curve analysis has been performed to test the accuracy of the regression model. A *P*-value < 0.05 was considered significant in all the calculations.

## Results

### Baseline features

The mean age of HS patients and controls was 43 ± 12 years and 46 ± 13, respectively (*P* = 0.16). Nearly half of the patients and controls were male. Thirteen HS patients and 12 controls were on statins at baseline (*P* = 0.68). One patient and 4 controls were taking ezetimibe (*P* = 0.16), and 2 HS patients were on fenofibrate (*P* = 0.16). There were no differences regarding the dose of statin between patients and controls (*P* = 0.83).

Patients with HS had significantly higher weight (82.4 ± 17.8 vs. 76.4 ± 16.9 Kg; *P* = 0.001), BMI (29.4 ± 5.4 vs. 26.5 ± 4.5 Kg/m2; *P* = 0.001), waist circumference (99.6 ± 14 vs. 91.4 ± 14 cm: *P* = 0.001), systolic blood pressure (132.6 ± 16.4 vs. 124.2 ± 15.8 mmHg; *P* = 0.003), diastolic blood pressure (82.0 ± 13.8 vs. 76.9 ± 8.3 mmHg; *P* = 0.012), HOMA-IR values (2.15 [1.00–3.73] vs. 1.48 [0.89–2.28]: *P* = 0.005), high-sensitive C-reactive protein –hs-CRP- (0.42 [0.17–0.89] vs. 0.10 [0.10–0.20]; *P* < 0.0001) and prevalence of smoking (65.4% vs. 19.4%; *P* < 0.0001), IR (46.2 vs. 19.4; *p* = 0.001) and metabolic syndrome (32.7 vs. 11.9; *P* = 0.004), than controls. Moreover, HS-patients had lower serum total cholesterol and HDL-C levels and higher AIP than the control group (Table 1).

**Table 1:** Lipid profile and atherogenic indexes in HS patients and controls

Parameter	HS patients (*n* = 78)	Controls (*n* = 62)	*P*
Total cholesterol, *mg/dL*	186.4 ± 33.6	202.7 ± 44.7	0.015
LDL-C, *mg/dL*	116.6 ± 32.4	122.4 ± 29.2	0.28
HDL-C, *mg/dL*	46.0 (41.0–56.3)	52.5 (46.8–69.5)	0.001
TG, *mg/dL*	87.5 (68.8–117.3)	74.0 (57.8–121.5)	0.23
Non-HDL cholesterol, *mg/dL*	138.5 (115.0–155.3)	136.5 (118.3–160.3)	0.45
ApoA1, *mg/dL*	146.0 (127.0–167.0)	151.0 (134.0–173.0)	0.12
ApoB, *mg/dL*	98.0 (79.5–112.3)	90.0 (77.8–104.3)	0.28
Lpa, *mg/dL*	15.0 (7.6–27.5)	15.5 (6.0–36.0)	0.83
Total cholesterol / HDL-C	4.09 (3.09–4.76)	3.51 (2.83–4.40)	0.06
LDL-C / HDL-C	2.49 (1.85–3.12)	2.19 (1.69–3.02)	0.09
Non-HDL-C / HDL-C	3.09 (2.09–3.76)	2.51 (1.83–3.40)	0.06
LDL-C / ApoB	1.22 (1.09–1.29)	1.35 (12.5–1.45)	< 0.0001
ApoB / ApoA1	0.65 (0.51–0.82)	0.60 (0.47–0.70)	0.09
AIP	−0.07 (− 0.24–0.05)	−0.23 (− 0.41–0.02)	0.016

*BMI* body mass index, *LDL-C* low-density lipoprotein, *HDL-C* high-density lipoprotein, *TG* triglycerides, *Lpa* lipoprotein (a), *AIP* atherogenic index of plasma

*Values are expressed as mean ± SD or median (interquartile range) as appropriate*

### Correlation between AIP and other variables

The correlations between AIP and several anthropometric and laboratory parameters, in cases and controls, are shown in Table 2. Noteworthy, AIP is significantly and positively related to BMI and waist circumference, systolic and diastolic blood pressure, and several lipid parameters, but also to HOMA and hs-CRP. Correlations were negative, as expected, with serum HDL-C and ApoA1 levels.

**Table 2:** Correlation between the atherogenic index of plasma and some demographic and laboratory variables in HS patients and controls

	HS patients (*n =* 78)		Controls (*n* = 62)	
	r	P	r	***P***
Age, *years*	0.233	0.049	0.210	0.10
BMI, *Kg/m*^*2*^	0.352	0.002	0.332	0.008
Waist circumference, *cm*	0.399	< 0.0001	0.349	0.005
Systolic BP, *mmHg*	0.231	0.042	0.277	0.03
Diastolic BP, *mmHg*	0.230	0.042	0.304	0.016
Total cholesterol, *mg/dL*	0.035	0.76	0.187	0.15
LDL-C, *mg/dL*	0.136	0.24	0.266	0.038
HDL-C, *mg/dL*	−0.655	< 0.0001	−0.709	< 0.0001
TG, *mg/dL*	0.884	< 0.0001	0.912	< 0.0001
Non-HDL cholesterol, *mg/dL*	0.380	0.001	0.554	< 0.0001
ApoA1, *mg/dL*	−0.408	< 0.0001	− 0.499	< 0.0001
ApoB, *mg/dL*	0.331	0.003	0.587	< 0.0001
Lpa, *mg/dL*	−0.080	0.48	−0.141	0.27
Fasting glucose, *mg/dL*	0.258	0.023	0.128	0.32
hs-CRP, *mg/dL*	0.343	0.002	0.362	0.004
HbA1c, *%*	0.375	0.001	0.181	0.16
HOMA index	0.551	< 0.0001	0.400	0.001
Insulin, *mIU/L*	0.491	< 0.0001	0.370	0.003

*BMI* body mass index, *BP* blood pressure, *LDL-C* low-density lipoprotein, *HDL-C* high-density lipoprotein, *TG* triglycerides, *Lpa* lipoprotein (a), *hs-CRP* high-sensitive C reactive protein*, HOMA* homeostatic model assessment

### AIP and HS severity

For the overall lipid profile analyzed, only AIP (*r =* 0.316; *P* = 0.005) was related to a more severe HS (PGA ≥ 3). After controlling for age, sex, BMI, IR, statin use, and active smoking this relationship persisted significant (*r =* 0.268; *P* = 0.023). No relationship between AIP and duration of HS was found. Table 3 shows the significant variables in HS patients according to the PGA score.

**Table 3:** Significant variables in HS patients according to the PGA score

Parameter	PGA < 3 (*n* = 32)	PGA ≥3 (*n* = 46)	*P*
Age, *years*	39.1 ± 11.6	45.1 ± 11.2	0.023
BMI, *Kg/m2*	28.0 ± 5.5	30.4 ± 5.2	0.043
Duration of HS, *months*	13.5 (5.3–22.5)	19.0 (9.8–27.3)	0.039
TG, *mg/dL*	71.0 (53.0–103.5)	93.0 (75.5–143.3)	0.003
AIP	−0.19 (−0.43–0.02)	−0.04 (−0.17–0.18)	0.006
hs-CRP, *mg/dL*	0.23 (0.12–0.58)	0.57 (0.31–1.06)	0.003
Fibrinogen, *mg/dL*	281.0 (264.5–324.3)	338.0 (289.0–392.8)	0.007

*BMI* body mass index, *LDL-C* low-density lipoprotein, *HDL-C* high-density lipoprotein, *TG* triglycerides, *Lpa* lipoprotein (a), *hs-CRP* high-sensitive C reactive protein, *AIP* atherogenic index of plasma

*Values are expressed as mean ± SD or median (interquartile range) as appropriate*

**Fig. 2:**
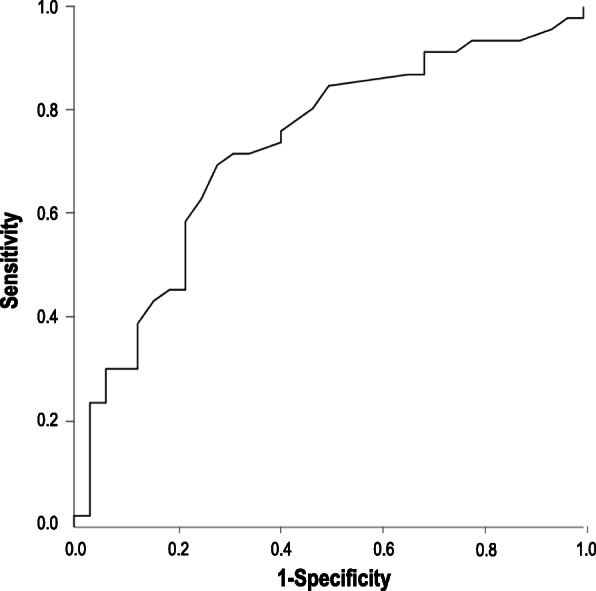
ROC curve for the regression model

The results of multiple logistic regression analysis of the parameters with potential association with HS severity, adjusted for age, sex, BMI, IR, smoking status, and statin use are shown in Table 4. Further adjustment for the duration of HS, hypertension, serum fibrinogen levels, and hs-CRP did not change these results. The exclusion of participants on statins (HS-patients and controls) yielded virtually identical results. Figure 2 shows the ROC curve for the regression model. The area under the curve is 0.73 (95%*CI*, 0.61–0.84; *P* = 0.001).

**Table 4:** Adjusted multiple logistic regression analysis showing the best set of factors associated with HS severity

	β-coefficient	OR (95%*CI*)	*P*
Age, *years*	0.050	1.05 (1.005–1.10)	0.03
AIP ≥ 0.11	1.476	4.38 (1.09–17.50)	0.037

*AIP* atherogenic index of plasma

## Discussion

Lipids are biological compounds that play multiple roles in human disease. Thus, neutral lipids, such as TG are critical in energy storage and have been involved in the pathogenesis of cardiovascular diseases, metabolic syndrome, and T2DM [22]. Lipid signaling pathways in patients with HS are poorly understood as well as the cellular mechanisms of lipid-mediated HS-induced pathogenesis. Gene expression of certain sphingolipids such as ceramide and sphingosine-1, that act as biologically active signaling molecules, have been implicated in the pathogenesis of the disease [23]. In a recent report, Fincher et al. [24], showed an increased localized accumulation of neutral lipids in HS-infected tissue as a result of a great bacterial load in these lesions. Moreover, recurrence of the disease has been linked to the development of dermic and subcutaneous sinus tracts, and lipids rafts in plasma membranes of keratinocytes also play a role in the regulation of metabolic and proliferative activity of these cells in HS patients [25]. Local steroidogenic activities in the skin have been implicated in the regulation of immune responses at local or systemic levels, and impaired of this cutaneous steroidogenesis has been linked to inflammatory skin disorders [26]. Finally, recent studies have shown that proprotein convertase subtilisin/kexin type 9 (PCSK9), a key regulator of lipid metabolism, may have pro-inflammatory activity on macrophages and has been linked to some markers of systemic inflammation, such as hs-CRP [27]. Nevertheless, there is no evidence to date, on its involvement in HS pathogenesis.

In clinical studies, HS patients often have higher serum TG and lower HDL-C levels than controls. The present study confirms a significant decrease in HDL-C in patients with HS compared with healthy controls. In this sense, Tsaousi et al. [28], found that matrix metalloproteinase 8, a collagen cleaving enzyme involved in the breakdown of extracellular matrix in normal and pathological processes, and in the degradation of ApoA1, a component of HDL particles, is one of the most highly upregulated molecules in HS lesions.

More recently, the AIP value, a logarithmically transformed ratio of molar concentrations of TG to HDL-C, has been reported as a good marker for the risk of atherosclerosis and cardiovascular disease [29]. AIP is an easily calculated parameter from the standard lipid profile that adds predictive value beyond that of the individual lipids and/or TC/HDL-C ratio. Furthermore, it is considered a subrogate of small LDL-C particle size distribution, with a better correlation than the LDL-C/ApoB ratio [30]. AIP provides additional information in predicting short- and long-term outcomes in patients with acute coronary syndrome but also it may be an independent factor for the risk of type 2 diabetes mellitus and metabolic syndrome [31, 32].

Interestingly, in HS patients (even after excluding the few participants on lipid-lowering agents) serum LDL-C, the traditional marker of the atherosclerotic burden was similar to controls. Nevertheless, AIP was higher in patients with HS than controls. In this sense, since HS is associated with high cardiovascular morbidity, AIP could be used as a good predictor of cardiovascular risk in these patients even in the presence of a normal lipid profile. Further studies on this matter would be interesting to perform.

In this study, AIP is related to BMI, waist circumference, blood pressure, lipid parameters, hs-CRP, and insulin resistance. Nevertheless, in the present study, AIP is an independent factor for the risk of a more severe HS, measured by the PGA. Besides, this association seems to be independent of hs-CRP, a well-known marker of inflammation. Besides, a cut-off point of 0.11 for this index has demonstrated to have a 4-fold increased risk for a PGA score ≥ 3. This index could be useful not only to detect patients at high risk for metabolic (obesity, diabetes, or metabolic syndrome) or cardiovascular complications (high blood pressure, cardiovascular events) but also to alert the clinician to the presence of a more severe HS.

### Study strength and limitations

This is the first study assessing the role of AIP in patients with HS. Nevertheless, it has the inherent limitations of a case-control study regarding causality. Besides, as an observational study, it may be subject to some bias due to the possible existence of confounders. However, to try to avoid this issue, adjustment for multiple potential confounding factors has been carried out.

## Conclusions

AIP is significantly and independently associated with a more severe HS. Therefore, clinicians might consider this index in the overall assessment of HS patients. Further large prospective studies are needed to confirm these results and explore the underlying mechanisms of the lipid-mediated HS-induced pathogenesis.

## Data Availability

The datasets generated and/or analyzed during the current study are not publicly available due to the protection of patients’ identity but are available from the corresponding author on reasonable request.

## Abbreviations

AIP: Atherogenic Index of Plasma
ApoA1: Apolipoprotein A1
ApoB: Apolipoprotein B100
BMI: Body Mass Index
BP: Blood Pressure
HDL-C: High-density Lipoprotein Cholesterol
HOMA: Homeostatic Model Assessment
HS: Hidradenitis Suppurativa
hs-CRP: High-sensitive C-Reactive Protein
IR: Insulin Resistance
LDL-C: Low-density Lipoprotein Cholesterol
Lpa: Lipoprotein (a)
PGA: Physician Global Assessment
PCSK9: Proprotein convertase subtilisin/kexin type 9
T2DM: Type 2 Diabetes Mellitus
TC: Total Cholesterol
TG: Triglycerides
TNF-α: Tumor Necrosis Factor-alpha
WC: Waist Circumference
